# Coping with Self-Threat and the Evaluation of Self-Related Traits: An fMRI Study

**DOI:** 10.1371/journal.pone.0136027

**Published:** 2015-09-02

**Authors:** Andreas Hoefler, Ursula Athenstaedt, Katja Corcoran, Franz Ebner, Anja Ischebeck

**Affiliations:** 1 Department of Psychology, University of Graz, Graz, Austria; 2 Neurosciences, BioTechMed Graz, Graz, Austria; 3 Department of Neuroradiology, Medical University of Graz, Graz, Austria; University of Medicine & Dentistry of NJ - New Jersey Medical School, UNITED STATES

## Abstract

A positive view of oneself is important for a healthy lifestyle. Self-protection mechanisms such as suppressing negative self-related information help us to maintain a positive view of ourselves. This is of special relevance when, for instance, a negative test result threatens our positive self-view. To date, it is not clear which brain areas support self-protective mechanisms under self-threat. In the present functional magnetic resonance imaging (fMRI) study the participants (N = 46) received a (negative vs. positive) performance test feedback before entering the scanner. In the scanner, the participants were instructed to ascribe personality traits either to themselves or to a famous other. Our results showed that participants responded slower to negative self-related traits compared to positive self-related traits. High self-esteem individuals responded slower to negative traits compared to low self-esteem individuals following a self-threat. This indicates that high self-esteem individuals engage more in self-enhancing strategies after a threat by inhibiting negative self-related information more successfully than low self-esteem individuals. This behavioral pattern was mirrored in the fMRI data as dACC correlated positively with trait self-esteem. Generally, ACC activation was attenuated under threat when participants evaluated self-relevant traits and even more for negative self-related traits. We also found that activation in the ACC was negatively correlated with response times, indicating that greater activation of the ACC is linked to better access (faster response) to positive self-related traits and to impaired access (slower response) to negative self-related traits. These results confirm the ACC function as important in managing threatened self-worth but indicate differences in trait self-esteem levels. The fMRI analyses also revealed a decrease in activation within the left Hippocampus and the right thalamus under threat. This indicates that a down-regulation of activation in these regions might also serve as coping mechanism in dealing with self-threat.

## Introduction

Individuals generally strive for a positive self-view and use all kinds of strategies to protect it [1,2]. These self-protective strategies will be activated when specific events threaten to impair a positive self-view. Such a threat may be a negative feedback concerning academic competence, social skills, or interpersonal relationships [3]. In the present functional magnetic resonance imaging (fMRI) study, we investigated the behavioral and neural consequences of self-protective strategies under threat. Participants had to evaluate self- and other related traits within the scanner immediately after receiving positive or negative performance-related feedback.

Keeping a positive self-view, the so called ‘self-positivity bias’, seems to be important for psychological well-being and dysphoric individuals lack this bias [4]. The *mnemic neglect model* proposes that negative self-related information is cognitively avoided [5] and thus less readily accessible from memory [6] than positive self-related information. This results in a positive self-view. People typically use more positive traits for self-description than negative traits [2]. Positive self-related information is also more easily (i.e. faster) retrieved from memory than negative self-related information [7].

Self-esteem is an important moderator when it comes to self-protection or self-enhancement [8]. High and low self-esteem individuals differ in the way they react towards self-threat, with high self-esteem individuals using more offensive strategies [9] in order to reestablish or heighten their self-esteem [1].

Neuropsychological research has recently scrutinized the neural bases of self-enhancement and self-protection [10]. In this research, activation within the anterior cingulate cortex (ACC) has been associated with the processing of negative self-related traits. Previous research could show an activation of the ventral anterior cingulate cortex (vACC) when negative traits had to be judged as being related to the self compared to a task requiring semantic processing [11]. There is also evidence for an attenuation of activation within the vACC when participants evaluated negative traits as being highly self-relevant compared to not so self-relevant traits [12]. Self-threat is also associated with a modulation of activation within the vACC, for example for a stereotype threat [13] or positive vs. negative social feedback [14,15]. The significance of the vACC for self-esteem protection is also supported by the association between hypometabolism in the vACC and unipolar depression [16]. Another ACC region that has been linked to self-threat reactions is the dorsal anterior cingulate cortex (dACC). Stronger activation in the dACC was observed during social exclusion compared to inclusion [17,18]. This is in line with the Sociometer theory [19] proposing that social exclusion is an existential self-threat and that self-esteem results from an inner monitor concerning the general social connectedness of an individual. Two other areas involved in the processing of self-threat are the medial prefrontal cortex (mPFC) [20] and the orbitofrontal cortex (OFC) [11,21]. The influence of trait self-esteem has also been investigated. The results suggest that only low self-esteem individuals but not high self-esteem individuals showed greater activation in the vACC and mPFC to positive versus negative social feedback [15,18].

To summarize, the evidence so far indicates the importance of the dACC and vACC in situations when individuals are confronted with a self-threat. For the vACC, the results point to an attenuation of activation under threat as activation was lower for individuals who found negative traits highly typical for themselves [12] as well as for individuals with unipolar depression [16]. This could mean that a threat triggers protective mechanisms that decrease the activation in the vACC. Different from the vACC, the dACC activation seems to increase under threat and could be associated with cognitive conflict and the distress individuals experience because of a self-threat.

In the present study, we threatened the state self-esteem of half of the participants with negative performance-related feedback. In the fMRI session immediately afterwards, participants had to evaluate either positive or negative traits by pressing a button indicating whether or not they applied to oneself (self-related) and or to the famous other (other-related). Similar to other fMRI- and behavorial studies [22,23,24] judgments on the self-descriptiveness of a trait were contrasted with judgments on how much the traits described another (famous) person. The present study investigated the interplay between self-related brain activity and self-concept accessibility after a self-threat. With this, the present study is similar to a previous study [22] where the interplay of self-threat on social comparison judgements was investigated. Different from the mentioned study [22] we were interested in differences between the processing of positive and negative self-related traits. We hypothesized that having to evaluate positive traits to the self gives individuals a possibility to enhance their self-concept after self-threat. In contrast, having to evaluate negative traits might even strengthen the effects of the threat. In line with the *mnemic neglect model*, we expected that individuals would react generally slower to negative self-related information then to positive self-related information. This difference should be larger after a self-threat than without a self-threat. Furthermore, we assumed that the reaction times on positive or negative traits might be correlated to activation in the vACC and dACC, as ACC activation seems to serve self-protective processes [10]. We also assumed that a negative performance feedback might especially affect individuals low trait self-esteem [8].

## Method

### Participants

Fourty-six participants, all of them students of the University Graz, took part in the experiment (24 female; *M* = 21.0 years; *SD* = 2.0 years). Participants were divided into four groups according to the two between-subjects factors (threat vs. no-threat and self-positive vs. self-negative) of the experiment. Their demographics are given in Table 1. They were recruited by an official service of the university-wide press office. All participants were German native speakers, right-handed and reported no health problems. After completing all study-relevant tasks the participants received a CD with their structural brain scan (T1) as compensation for their participation.

**Table 1:** Demographics of the threat vs. no-threat group who evaluated either self-positive/other-negative or self-negative/other positive traits.

	threat	no-threat
self-positive/other negative	12 (5 male) age 21.2 (*SD* = 2.3)	11 (5 male) age 21.3 (*SD* = 2.0)
self-negative/other positive	10 (5 male) age 21.1 (*SD* = 2.7)	13 (7 male) age 21.3 (*SD* = 1.6)

### Ethics statement

The study was approved by the Ethics Committee of the Medical University Graz (EK no. 21–376 ex 09/10). Participants completed a consent form at the beginning of the experiment. The consent form did not state the true aim of the experiment. The participants received a detailed debriefing afterwards. This debriefing was an integral part of the ethics application.

### Materials of the fMRI task

Within the scanner, adjectives and short trait expressions, such as ‘friendly’, were presented and the participants were instructed to judge whether they agree or disagree with these expressions (Yes/No) when asked to relate them either to themselves (‘Self’ condition) or to a famous politician (Angela Merkel or Nikolas Sarkozy, ‘Other’ condition). We used a list of 120 personality traits that consisted of 60 positive and 60 negative short phrases or adjectives, such as, for example, *friendly* for a positive trait and *forgetful* for a negative trait. There were 40 traits (20 positive and 20 negative) representing the BIG-FIVE personality dimensions [25] and 40 positive traits stemming from a gender-typicality scale [26]. Finally we included 40 negative traits from previous gender stereotypes research [27]. The list of personality traits was randomized. Participants were asked to press one of two buttons on a button box with their right hand. Response latencies were recorded as an indicator of self-concept accessibility: it is assumed that faster response latencies reflect higher accessibility [7,28]. Table 1 shows the demographics of the participants in the four groups.

### Procedure

After completing the informed consent form, the participants had to answer the *Rosenberg Self-Esteem scale* (for a german validation) [29]. This scale measures the habitual or trait self-esteem with five positive (e.g. “*On the whole*, *I’m satisfied with myself”)* and five negative items (e.g. “*I certainly feel useless at times”)*. Participants had to rate their agreement on a four-point rating scale (from 0 = strongly disagree to 3 = strongly agree). The German version of the Rosenberg scale has a Cronbach’s α = .81, indicating high reliability. In addition, Table 2 shows the evenly distribution of trait self-esteem over the four participant groups.

**Table 2:** Means of Trait self-esteem sum scores of the four participant groups.

	threat	no-threat
self-positive/other negative	25.25	22.36
self-negative/other positive	21.70	24.23

Mean trait self-esteem sum scores were gained from the Rosenberg scale.

Sums were calculated over the items of the scale. There were no significant differences.

Before entering the scanner participants were asked to complete a test of general cognitive performance (WPT, Wonderlic Personell Test) [30] within eight minutes. The WPT consists of 50 items which require basic arithmetic knowledge and general problem solving skills. The typical time limit of 12 minutes was shortened to 8 minutes to render a complete solution of the test unlikely.

Immediately before starting the scanner, half of the participants received a positive (no-threat condition), half a negative performance feedback (self-threat condition) independently of their performance. For positive feedback, participants were told that their results were above average. This positive feedback was considered neutral as it is consistent with a student’s academic self-concept to perceive themselves as being of above-average intelligence. For negative feedback, participants were told that their results were below average.

**Fig 1:**
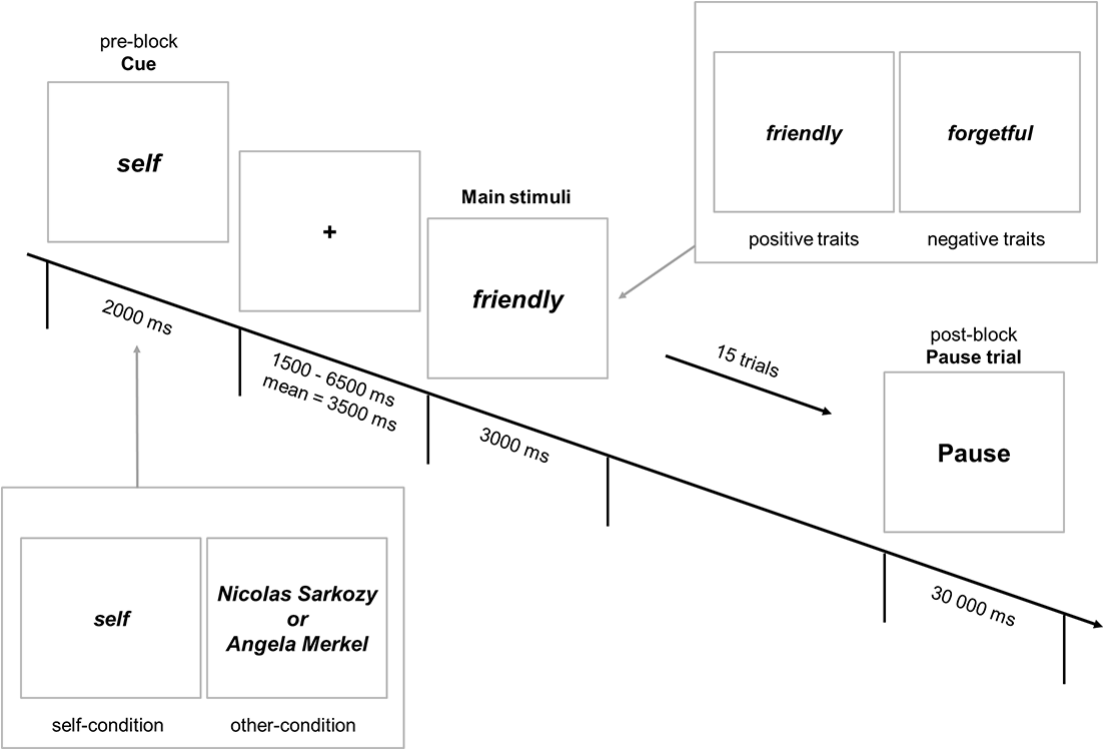
Experimental procedure within the scanner. Each block began with the display of the cue (“self” or “Nikolaus Sarkozy”) for 2000 ms. A fixation cross was displayed with a mean duration of 3500 ms. Then either a positive or a negative personality trait was shown for 3000 ms. The response (agree or disagree) had to given within a 3000 ms time limit.

Within the scanner, participants had to evaluate personality traits either referring to the self or another, famous person. Eight blocks were presented in total. The conditions (self/other) as well as the personality traits were blocked with regard to valence. One half of the participants received self- and the other half other-related traits as a first block. Every trait was presented once, resulting in 15 traits per block. Each block began with the presentation of the reference (e.g., ‘Angela Merkel’ or ‘Self’) as the cue information. This lasted 2000 ms. In the following, a fixation cross was presented for a jittered time interval between 1500 and 6500 ms (mean duration: 3500 ms). The fixation cross was followed by the first trait of the first block for 3000 ms. Participants had to respond within the time limit otherwise the trial was rated as a time out. The participants’ task was to respond by pressing one out of two buttons on a button box with their right hand. They responded with their fore finger if they agreed and with the middle finger if they disagreed with the presented trait, depending on the reference (self vs. other). To control for gender effects, the reference *other* was for male participants ‘Nicolas Sarkozy’ and for females ‘Angela Merkel’. After finishing a stimulus block, a cue for a resting block (Pause) was displayed on the screen for 30 seconds. The second stimulus block started with the cue information (evaluate for 'Self' or 'Sarkozy' / 'Merkel'). The main experiment lasted for 20 minutes on average. Mean response latencies were collapsed over agree and disagree responses but separately for positive and negative traits. They were taken as indicators for self-concept activation [28]. For the presentation of the stimuli and the synchronization with the scanner *Presentation* software (Version 14.8, Neurobehavioral Systems) was used. Fig 1 shows the temporal organization of the task within the scanner.

After scanning, participants had to complete an explicit state self-esteem questionnaire, to assess their state self-esteem level as a manipulation check with respect to the self-threat received directly before the scanning session. We used the *State Self-Esteem Scale* (SSES) [31] which consists of three different scales representing different aspects of self-esteem (performance, social, appearance scale). We had translated the 7-items *performance scale* from the 20-items questionnaire into German. Examples for items loading on the performance factor are *I feel that I am not doing well* or *I feel frustrated and rattled about my performance*. The participants had to rate their agreement on a 6-point rating scale (from 1 = *not at all like me* to 6 = *most like me*). The SSES values of the performance scale were summed to yield the SSES score. After the translation the reliability of the questionnaire (Cronbach’s α = .72) was similar to the English version. Finally, all participants were debriefed. All of them stated to have taken the false performance feedback seriously.

### Functional imaging

The fMRI was performed using a 3.0-T Tim Trio System scanner (Siemens Medical System; Erlangen, Germany). First, an anatomical image was recorded using a T1-weighted 3D MPRAGE sequence (TR = 1900 ms, TE = 2,19 ms, isotropic resolution was 1 x 1 x 1 mm). In total, 227 functional scans were recorded (the first two scans were discarded automatically by the scanner). All scans were recorded with a single shot gradient-echo EPI pulse sequence. Each volume consisted of 31 transverse slices in descending order, with a slice thickness of 3 mm. The time interval between the acquisitions (TR) was 2000 ms, the echo time (TE) was 24 ms with a flip angle of 90°. The field of view (FoV) was 192 mm^2^. With a matrix size of 64 x 64 the in-plane resolution was 3 x 3 x 3 mm. The functional scan was started with the beginning of the experimental paradigm.

### Preprocessing of behavioral data

In a first step, response latencies above or below (±3 SD) the mean of the adjectives were excluded from further preprocessing procedure [32]. Response latencies were transformed to correct for the number of syllables, by dividing the total reaction latency by the number of syllables in a trait expression (e.g. latencies for *friendly* were divided by 2). Finally these values were transformed using the natural logarithm (ln). The transformed response latencies were used for correlational analyses of the regions of interests (ROI) and further behavioral analyses.

### Statistical analysis

Preprocessing and statistical analysis of the fMRI data was performed with SPM8 (Wellcome Department of Cognitive Neurology, London UK) software for MATLAB (Version R2010a, The MathWorks Inc., Natick, MA). After the DICOM import, the first two images of every fMRI run were discarded manually, to ensure signal stabilization Together with the two automatically discarded images this means that the first four images of the time series were removed. After motion correction (realigning and unwarping the images), slice time acquisition correction and normalization were performed. Finally, the functional images were smoothed with a Gaussian kernel of 8 mm FWHM. Normalization of the functional fMRI images was checked manually.

For the first level analysis, an event-related model with three conditions (self, other, fixation) was analyzed. The delta-function of the trial onsets for each condition was convolved with the canonical form of the hemodynamic response function. The onset of the cue as well as the six motion parameters from the realignment procedure were entered into the model as parameters of no interest. A high-pass filter (cut-off frequency: 1/500 Hz) was used to remove low frequency drifts. No global normalization was used. The contrasts *self > other* were calculated per subject and entered into a group analysis (second level or random effects analysis) [33]. To compare the different groups (threat vs. no-threat, positive vs. negative valence) a factorial design was used. The resultant statistical parameter maps were thresholded using an initial uncorrected p-value threshold of less than 0.001, reporting only clusters as significant that had a corrected p-value of less than 0.05 on cluster level (FWE corrected).

#### ROI-analysis

To investigate differences within specific anatomical regions more closely, a region of interest (ROI) analysis was performed. This analysis was based on the combined right and left ACC-ROIs mapped within the MATLAB Toolbox ‘AAL’ (Anatomical Automatic Labeling, Cyceron). The extent of the ACC in the z-Coordinate was halved to generate two ROIs, one for the dorsal part (dACC) and one for the ventral part (vACC). For the ROI analyses, the MATLAB Toolbox ‘Marsbar’ was used (M. Brett, http://marsbar.sourceforge.net). The ROI analyses were calculated using the contrast values of each condition against baseline averaged over all voxels of the ROI per participant. Averaged contrast values per participant and condition were then entered into an analysis of variance. Contrast values have the advantage that they can be directly entered into a second level statistical analysis [34]. For the figures, we calculated percent signal change using the marsbar toolbox.

## Results

### Manipulation check

**Fig 2:**
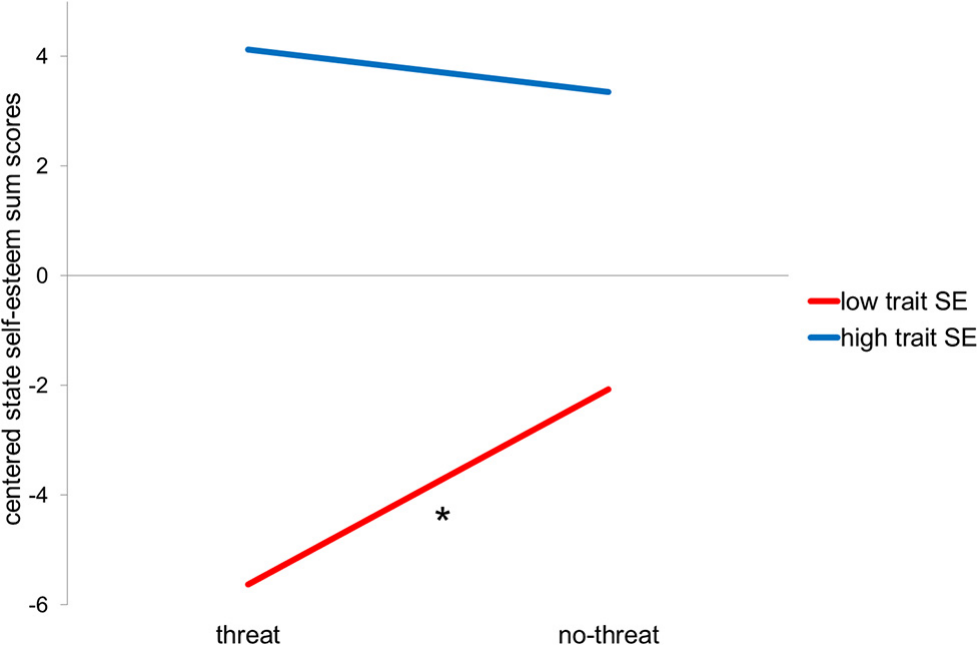
Regression plot of the manipulation check analyses. Centered SSES sum scores as the criterion (y-axis), the performance feedback condition as the predictor (x-axis) and separated by continuous trait self-esteem scores as the moderator (levels are calculated automatically as ±1 SD below and above mean). * Significance was tested on a p<.05 level.

We first computed a manipulation check by calculating a multiple regression based moderator analysis with state self-esteem as the criterion and feedback (threat vs. no-threat) and trait self-esteem (as continuous variable) as predictors. The results revealed a significant main effect of trait self-esteem (*b* = 7.95, *t*_43_ = 7.35, *p* = .000), which was driven by a significant interaction between the two predictors trait self-esteem and feedback (see Fig 2; *b* = -4.59, *t*_43_ = -2.11, *p* = .041) reflecting that state self-esteem decreased after receiving a negative feedback compared to positive feedback but only for low self-esteem individuals (*t*_43_ = 2,47, *p* = .018). These result suggests, that the manipulation check was not successful for high self-esteem individuals (*t*_43_ = -.54, *p* = .596). However, it should be mentioned that state self-esteem was measured after the scanning session outside of the scanner and that further analyses (see below) indicated that high self-esteem individuals dealt more successfully with the threat than low self-esteem individuals during the scanning session.

### Behavioral results

To investigate differences concerning cognitive trait accessibility we undertook two steps of analysis for the transformed response latencies. First, in order to check for accessibility differences between self- and other-related stimuli, we computed an ANOVA on the transformed response latencies as dependent variable. *Reference* (self vs. other) was included as within-subjects factor and both *feedback* (threat vs. no-threat) and *presentation order* (self-reference first vs. other-reference first) as between-subjects variables. This analysis revealed only one significant main effect for *reference* (*F*_1,42_ = 8.046, MSE = .015, *p* = .007) showing that participants processed self-related traits faster (*M* = 6.13, SD = .19) than other-related traits (*M* = 6.21, SD = .16).

In the second step, we analyzed self- and other-related responses separately in order to examine the relevance of the trait valence. In both analyses, we included three between-subjects factors, i.e. *valence* (positive vs. negative), *feedback* (threat vs. no-threat), and presentation order (positive first vs. negative first). For self-related traits, we found a significant main effect only for *valence* (*F*_1,38_ = 55.402, MSE = .016, *p* = .000) indicating that individuals responded faster to positive self-related traits (*M* = 5.99, SD = .13) than to negative self-related traits (*M* = 6.28, SD = .12). For the other-related traits, we found no significant effects.

**Fig 3:**
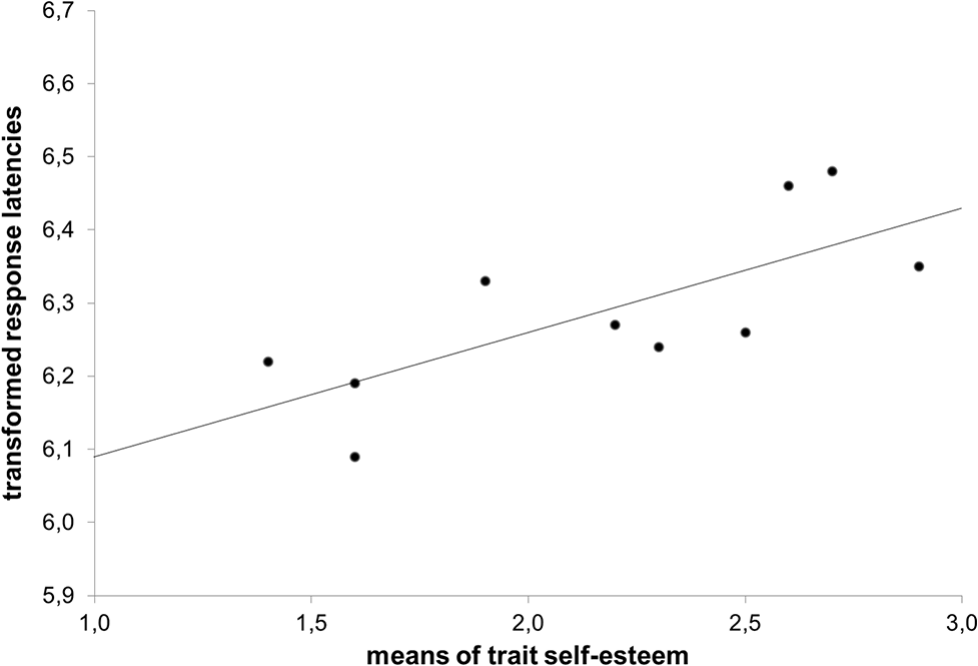
Scatter plot of the significant correlation between the log-transformed reaction times of the negative self-related traits and trait self-esteem under self-threat.

As trait self-esteem had been a moderator in relation with self-threat, we wanted to know if there was any link between trait self-esteem and self-concept activation. We calculated correlations between self-related trait response latencies and trait self-esteem separately for participants with and without self-threat (see Table 3). We found a significantly positive correlation for threatened participants between response latencies for negative self-related traits and trait self-esteem. Individuals with high trait self-esteem responded slower to negative traits compared to low self-esteem individuals (*r* = .74; *p* = .020, see also Fig 3 for a scatter plot). This result suggests that negative self-related traits were less accessible for individuals with high trait self-esteem than for low self-esteem individuals.

**Table 3:** Correlations between trait self-esteem and the self-related traits response latencies separately for participants with and without self-threat.

	positive self-related traits	negative self-related traits
**self-threat**	-0.20	.74*
**no-threat**	-.45	.17

* for *p <*. *05*.

### fMRI results

**Fig 4:**
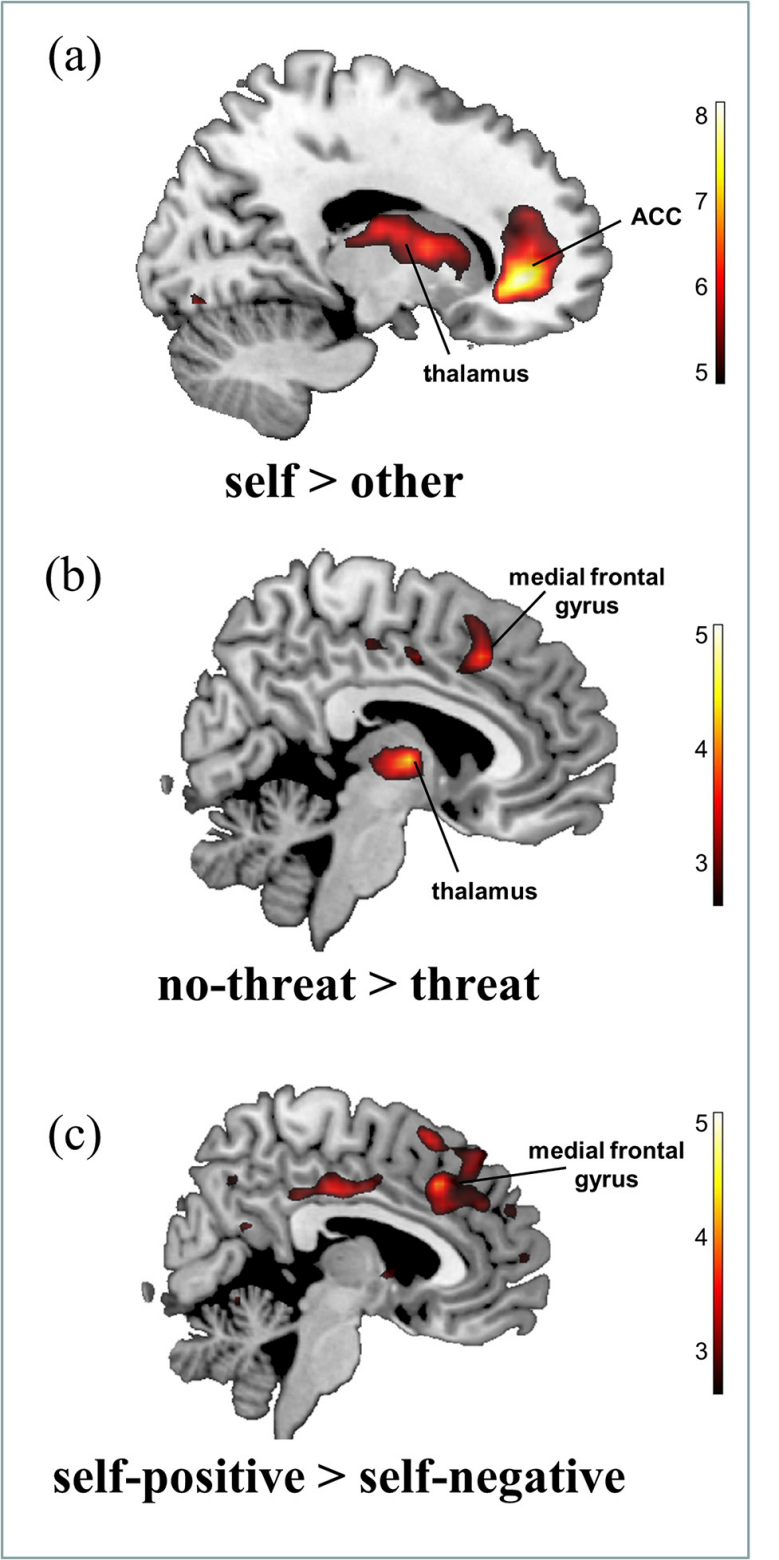
Group activation differences, whole brain analysis: a) activations for the within-subjects contrast *self > other*. b) Activations for the between-subjects contrast *no-threat > self-threat* (negative feedback on the intelligence test result) c) Activations for the between-subjects contrast *positive traits > negative traits* (self-reference only). All activations are shown for a threshold of *p* < 0.001 uncorrected, reporting only clusters that have *p* < 0.05 corrected on cluster level.

We first analyzed the within-subjects contrast between processing self- vs. other-related traits. The analyses revealed significant activations in midline cortical areas, including the ACC and the thalamus for self-related traits compared to other-related traits. An overview of the activated areas is given in Table 4 and Fig 4A, whereas the associated coordinates are reported as given by SPM8 (MNI space), corresponding approximately to Talairach & Tournoux space [35,36].

**Table 4:** Within-subjects group differences in activation: self-related vs. other-related activation (namely contrast: *self>other*) and other-related vs. self-related activation (namely contrast: *other>self*).

Side	Area	x	y	z	K	Z
	**self > other**					
Left	Anterior Cingulate Cortex	-6	38	1	1017	7.25
Right	Thalamus	6	-4	7	1017*	6.11
	**other > self**					
	Ns.					

Statistical parameter maps were thresholded at Z > 5 uncorrected, reporting only clusters with more than 30 voxels. k = cluster size, Z = Z value for the maximally activated voxel of the cluster.

*The activation is part of a bigger cluster.

To investigate the differences with regard to the main effects of *threat* and *valence* in this contrast, we evaluated group differences in a 2x2 factorial design with the factors *threat* (self-threat vs. no-threat) and *valence* (positive vs. negative).

#### Main effects

For the contrast *no-threat* > *threat*, activations were observed within the left Hippocampus, the right thalamus, and the right ACC/medial frontal gyrus. This could indicate that these regions were under-activated in a self-threat situation. There was no significant activations in the contrast *threat* > *no-threat*. In the contrast *positive traits > negative traits*, activations were observed within the left ACC, the middle cingulate cortex as well as the left and right angular gyrus. There was no activation for the contrast *negative traits > positive traits*. The results for the main effects are given in Table 5 and Fig 4B and 4C.

**Table 5:** Between-subjects group differences in activation: self-threat and no-threat activation (contrasts: *threat>no-threat* and *no-threat>threat*); activation for positive self-related and negative self-related traits (contrasts: *self-positive>self-negative* and *self-negative>self-positive*).

Side	Area	x	y	z	K	Z
	**threat > no-threat**					
	ns.					
	**no-threat > threat**					
Left	Hippocampus	-39	-28	-2	253	4.26
Right	Thalamus	3	-7	4	445	4.03
Right	Middle Cingulate Gyrus	6	20	43	258	3.59
	**self-positive > self-negative**					
Left	Anterior Cingulate Cortex	-6	38	25	1697	4.65
Right	Angular Gyrus	48	-64	34	270	4.63
Left	Angular Gyrus	-51	-55	37	406	3.88
Left	Middle Cingulate Gyrus	-6	-34	37	262	3.68
	**self-negative > self-positive**					
	ns.					

Statistical parameter maps were thresholded at p < 0.005 uncorrected, reporting only clusters with p < 0.05 corrected on cluster level. k = cluster size, Z = Z value for the maximally activated voxel of the cluster.

### ROI-analyses

**Fig 5:**
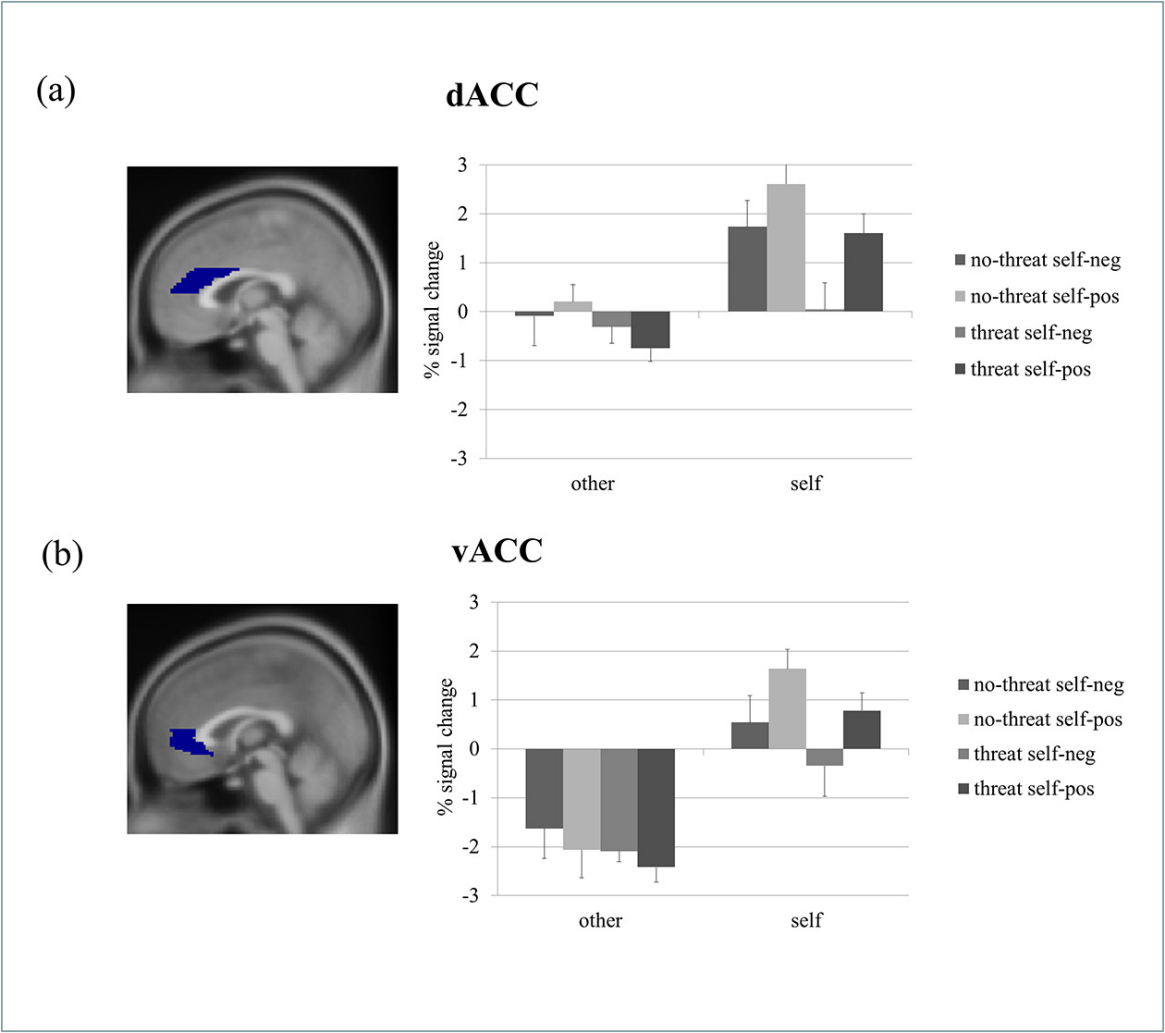
ROI-analyses: Averaged percent signal change values for all conditions against baseline shown for a) the dorsal anterior cingulate cortex and b) the ventral anterior cingulate cortex.

To further evaluate the results of the whole-brain fMRI analysis in the light of the literature reviewed, an ROI analysis of the ACC (dACC and vACC) was conducted. In a first step, we calculated a repeated measurement ANOVA with the between-subjects factors *threat* (threat vs. no-threat), *valence* (positive vs. negative), and *self-esteem group* (high vs. low), as well as the within-subjects factors *reference* (self vs. other) and the two ROIs. Self-esteem group (generated by a median split) did not influence the ROI results; there was neither a significant main effect nor any significant interaction with self-esteem group. Effect sizes depended on ROI, however, leading to a significant main effect (*F*_1,37_ = 99.89, *MSE* = 6905.83, *p* = .001), a significant interaction with the factor reference (*F*_1,37_ = 42.52, *MSE* = 812.93, *p* = .001) and a significant quadruple interaction (*F*_1,37_ = 6.39, *MSE* = 122.15, *p* = .016) of the factors *ROI*, *threat*, *valence*, and *reference*. Consequently in a next step, we analyzed both ROIs separately but removed *self-esteem group* as a factor. The mean activation values (percent signal change) per condition are shown in Fig 5A for the dACC and Fig 5B for the vACC.

For the dACC, the main effect of *threat* was significant (*F*_1,41_ = 5.74, MSE = 2046.97, *p* = .021), with greater activation in the no-threat condition compared to the threat condition. The main effect of *reference* was also significant (*F*_1,41_ = 51.47, MSE = 7616.15, *p* = .000) showing more activation during evaluation of self-related traits compared to other-related traits. Finally, the analysis revealed an interaction between *reference* and *valence* (*F*_1,41_ = 4.80, MSE = 710.33, *p* = .034). Activation for evaluating negative traits related to the self was lower than for positive traits related to the self. No such difference could be found for other-related traits. Fig 5A shows for individuals responding to self-related traits the lowest dACC activation for negative traits. This can be seen as an additive effect in dependence of the two main effects.

For the vACC, the analysis revealed also a significant main effect of *reference* (*F*_1,41_ = 95.48, MSE = 15584.83, *p* = .000) and an interaction *reference* and *valence* (*F*_1,41_ = 4.18, MSE = 681.85, *p* = .047). These effects can be interpreted the same way as those for the dACC analyses. The main effect of threat was not significant.

#### Correlations between self-concept, trait self-esteem, and brain activation

**Fig 6:**
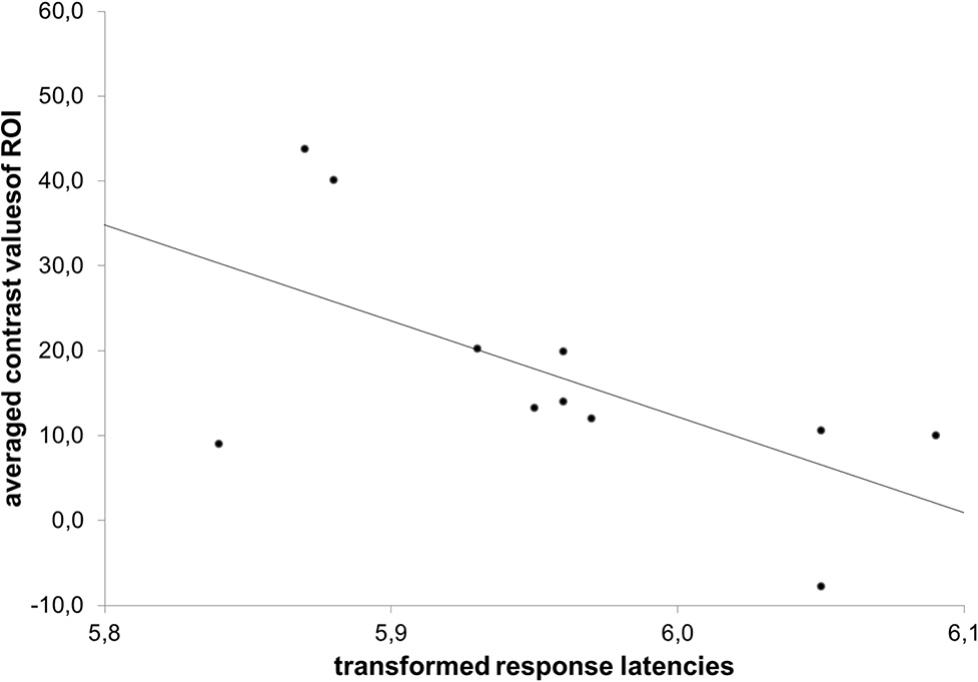
Correlation with ROI contrast values: the scatter plot demonstrates the significant correlation between averaged ROI contrast values of dACC activation and the associated log-transformed means of response latencies of the positive self-related traits.

We correlated the trait response latencies with the activation in the two regions of interest, namely, the dorsal and the ventral part of the ACC. Specifically, we examined the link between brain activation and positive and negative self-related trait latencies under threat vs. no-threat (see Table 5, upper part and scatter plot in Fig 6). For individuals in the threat condition, we found a significant negative correlation between the response latencies for positive self-related traits and the dACC activation (*r* = -.63, p = .038) and and a similar but not significant correlation for the vACC activation (*r* = -.55, p = .078). Individuals responded faster to positive self-related traits under self-threat the more the dACC (and the vACC regions) was activated. In contrast, the respective correlations for the negative self-related traits were positive, although both not significant. The direction of this correlation indicates that under self-threat individuals responded slower to negative self-related traits the more the ACC was activated. This pattern of correlations suggests that the ACC plays an important role in managing self-related information processing and protecting self-esteem under threat.

Finally, we correlated trait self-esteem with dACC and vACC activation (Table 6). There was a considerable but not significant correlation between dACC activation and trait self-esteem after self-threat (*r* = .40; *p* = .071) indicating that the higher the individuals’ trait self-esteem level, the more the dACC was activated after self-threat.

**Table 6:** Correlations between ROI activation scores and response latencies for self-concept activation and trait self-esteem scores under different conditions (threat vs. no-threat): dACC activation (dorsal anterior cingulate cortex) or vACC activation (ventral anterior cingulate cortex).

		ACC activation	vACC-activation
**positive traits**	self-threat	-.62*	-.55
	no-threat	.06	.39
**negative traits**	self-threat	.43	.46
	no-threat	-.49	-.29
**trait self-esteem**	self-threat	.40	.19
	no-threat	-.16	.01

* for *p <*. 05.

## Discussion

The present study aimed at gaining more insight into the neural basis of the cognitive processes involved in self-concept activation following a self-threat. We threatened the self-esteem of half of our participants by inducing a negative test performance feedback immediately before they entered the scanner. In the scanner they were asked to ascribe positive or negative traits either to themselves (self-related) or to a famous other (other-related). We found a greater activation in the ACC for self- rather than other-related traits. In contrast to our expectations, ACC activation was higher without threat compared to the threat condition and for evaluations of positive than for negative self-relevant traits. The ROI-analysis showed, that the strongest effects of threat and valence were observed when individuals evaluated self-related traits. We see this result in line with the research of [12] who also found vACC attenuation when individuals judged negative traits to be highly self-descriptive. We infer that self-threats trigger a protective mechanism that attenuates the activation in the vACC and dACC.

A closer look at the correlation analyses we reported gives more clarity about the function of the ACC regions. Individuals reacted faster to positive self-related traits the more the ACC was activated. This result indicates that the ACC might play an important in re-establishing self-esteem.

The correlational analyses and the ANOVA results reveal a complicated functional relation as, on the one hand, ACC activation was shown to be attenuated after self-threat and, on the other hand, it was linked to self-esteem regulation under self-threat. It is possible that individuals who showed less inhibition showed a greater variability in relating positive and negative traits to the self. The additional analyses that we performed for trait self-esteem suggest that high self-esteem individuals were the ones who showed this self-esteem regulation. First, high self-esteem individuals responded slower to negative traits after a self-threat (and faster to positive traits). Based upon cognitive self-concept theories we propose that the time to evaluate self-related traits mirrors self-knowledge accessibility [7]. Consequently, high self-esteem individuals might have inhibited the accessibility of negative self-knowledge after a self-threat whereas they enhanced the accessibility of positive self-knowledge. This result is also in line with the *mnemic neglect model* [5] and confirms our expectations that differences in reaction times for negative and positive traits should be larger after self-threat at least for high self-esteem individuals. Second, high self-esteem individuals tended to have higher dACC activation after self-threat than low self-esteem individuals. We propose that our results point towards self-enhancing strategies of high self-esteem individuals after a self-threat [1]. This is in line with research showing that high self-esteem individuals use more *offensive* strategies to self-regulate [9]. Our results could also explain why only low self-esteem individuals were effectively threatened in the manipulation check after the scanning session. It is possible that high self-esteem participants had already worked through the threat by activating positive and ignoring negative self-related traits. Our results point to a disadvantage of the observed attenuation of ACC activation after self-threat as this might also impair the participants' ability to overcome the threat.

Our study shows similar results for the two brain regions of interest, the vACC and the dACC. In this, our study is different to existing literature which usually shows the involvement of either the one or the other region [11,12,13,17,18]. It should be noted, however, that this study is the first that used a clearly defined anatomical ROI. Our results fit well with the functional interpretation for the dACC, as this brain region might reflect cognitive conflict and the distress individuals experience due to self-threat. Our results also indicated that the dACC is involved in active self-enhancement strategies. Taken together, our results are in line with earlier research showing ACC self-threat involvement [10,11,12,13,17,18].

The fMRI analyses also revealed a decrease in activation within the right thalamus under threat. This could be interpreted as a diminished state of attention. Thalamus activation has been associated with attention processes, for instance, processes that achieve and maintain an attentional state of alertness [37] or attention processes that mediate attention and arousal [38]. Importantly, the thalamus is also assumed to fulfill a filter function given its crucial role as a gateway of sensory information to the cortex and to consciousness. In fact, it is assumed that the so-called *fronto-thalamic gate system* is involved in regulating attentional intensity [39]. Sensory input can be modulated or blocked while being processed by the thalamic nuclei [40]. The thalamus is therefore often described as a *center of regulation* or *gate of consciousness* in information processing [41]. This functional role of the thalamus might play a role when coping with self-threat. It could be shown that the thalamus is more involved in negative compared to positive self-related processing [42]. Shutting down thalamic activation after self-threat might help in tuning out negative self-relevant information processing on a very early level. However, this interpretation is speculative and should be further researched.

To conclude, the present study is one of the first that investigated the neural basis of the interplay between self-threat reactions and self-enhancing processes following a self-threat (but see also [21]). Our results showed ACC attenuation following a self-threat. They also confirm the role of the dACC in managing the accessibility of self-related information. Faster processing of positive self-related traits and slower processing of negative self-related traits after self-threat was correlated with more dACC activation, as well as with trait self-esteem. The activation of the dACC might therefore measure participants' resilience to threat. Secondly, the attenuation of the dACC and the thalamus under threat suggests a protective mechanism that regulates the activation in both brain areas. However, the mechanism might also impair dealing successfully with the threat. The evidence matches with the assumptions of the *mnemic neglect model* [5]. In this model self-threatening feedback is processed superficially whereas self-affirming feedback is processed more thoroughly. Finally, our study showed that trait self-esteem is a potential variable that moderates the applied coping strategy as well as the corresponding activation pattern in the ACC. A main goal of future research is to further elucidate the functional significance of the entire ACC in the protection against threat and its role in an individual's resilience. A further question will also be to get better insight into the brain's protective mechanisms against unfavorable and potentially threatening information. It is possible that information is already blocked at an early stage as suggested by the deactivation of the thalamus under threat.
